# Ultrasonic dissection versus electrocautery dissection in laparoscopic cholecystectomy for acute cholecystitis: a randomized controlled trial (SONOCHOL-trial)

**DOI:** 10.1186/s13017-024-00565-4

**Published:** 2024-11-13

**Authors:** My Blohm, Gabriel Sandblom, Lars Enochsson, Yücel Cengiz, Haytham Bayadsi, Joakim Hennings, Angelica Diaz Pannes, Erik Stenberg, Kerstin Bewö, Johanna Österberg

**Affiliations:** 1 Department of Clinical Science and Education, Södersjukhuset, Karolinska Institutet, Stockholm, Sweden; 2 Centre for Clinical Research Dalarna, Uppsala University, Falun, Sweden; 3 Department of Surgery, Mora Lasarett, Mora, Sweden; 4 Department of Diagnostics and Intervention, Surgery, Umeå University, Umeå, Sweden; 5 Division of Orthopedics and Biotechnology, Department of Clinical Science, Intervention and Technology, Karolinska Institutet, Stockholm, Sweden; 6 Department of Surgery, Södertälje Hospital, Södertälje, Sweden; 7 Department of Surgery, Faculty of Medicine, and Health, Örebro University Hospital, Örebro, Sweden; 8 Department of Surgery, Falu Lasarett, Falun, Sweden

**Keywords:** General surgery, Acute care surgery, Acute cholecystitis, Minimally invasive surgical procedures, Laparoscopic cholecystectomy, Electrosurgery, Ultrasonic surgical procedures, Electrocoagulation

## Abstract

**Background:**

Laparoscopic cholecystectomy with ultrasonic dissection presents a compelling alternative to conventional electrocautery. The evidence for elective cholecystectomy supports the adoption of ultrasonic dissection, citing advantages such as reduced operating time, diminished bleeding, shorter hospital stays and decreased postoperative pain and nausea. However, the efficacy of this procedure in emergency surgery and patients diagnosed with acute cholecystitis remains uncertain. The aim of this study was to compare outcomes of electrocautery and ultrasonic dissection in patients with acute cholecystitis.

**Methods:**

A randomized, parallel, double-blinded, multicentre controlled trial was conducted across eight Swedish hospitals. Eligible participants were individuals aged ≥ 18 years with acute cholecystitis lasting ≤ 7 days. Laparoscopic cholecystectomy was performed in the emergency setting as soon as local circumstances permitted. Random allocation to electrocautery or ultrasonic dissection was performed in a 1:1 ratio. The primary endpoint was the total complication rate, analysed using an intention-to-treat approach. The primary outcome was analysed using logistic generalized estimated equations. Patients, postoperative caregivers, and follow-up personnel were blinded to group assignment.

**Results:**

From September 2019 to March 2023, 300 patients were enrolled and randomly assigned to electrocautery dissection (n = 148) and ultrasonic dissection (n = 152). No significant difference in complication rate was observed between the groups (risk difference [RD] 1.6%, 95% confidence interval [CI], − 7.2% to 10.4%, *P* = 0.720). No significant disparities in operating time, conversion rate, hospital stay or readmission rates between the groups were noted. Haemostatic agents were more frequently used in electrocautery dissection (RD 10.6%, 95% CI, 1.3% to 19.8%, *P* = 0.025).

**Conclusions:**

Ultrasonic dissection and electrocautery dissection demonstrate comparable risks for complications in emergency surgery for patients with acute cholecystitis. Ultrasonic dissection is a viable alternative to electrocautery dissection or can be used as a complementary method in laparoscopic cholecystectomy for acute cholecystitis.

**Trial registration:**

The trial was registered prior to conducting the research on http://clinical.trials.gov, NCT03014817.

**Supplementary Information:**

The online version contains supplementary material available at 10.1186/s13017-024-00565-4.

## Background

Laparoscopic cholecystectomy by ultrasonic dissection is an established alternative to traditional monopolar electrocautery dissection. Previous research on elective cholecystectomy supports the use of ultrasonic dissection because of its numerous benefits, including reduced operating time, diminished bleeding, fewer gallbladder perforations, shorter hospitalization and decreased postoperative pain and nausea [1–11]. Comparable outcomes have also been demonstrated [12, 13]. Despite these advantages, counterarguments to widespread adoption include increased instrumental costs and challenges in instrument handling during the learning curve. Therefore, the preferred instrument for most surgeons continues to be monopolar electrocautery.

The unique features of the ultrasonic instrument —allowing for both cutting and coagulation for bleeding control, as well as tissue sealing and vaporization capacities —offer theoretical advantages in emergent surgical settings involving acute cholecystitis, where hyper-vascularized, oedematous tissue and omental adhesions are common [14]. These capabilities may provide notable benefits during acute cholecystectomies. However, evidence supporting its use in emergency surgery is sparce, with only a few small studies published on its application in acute cholecystitis. For instance, a randomized single-centre study of 42 patients with acute cholecystitis reported fewer conversions and reduced blood loss using ultrasonic dissection [15]. Additionally, subgroup analyses of intraoperatively diagnosed acute cholecystitis in elective studies have demonstrated shorter operating times [2]. However, whether ultrasonic dissection decreases intra- and postoperative complications remains uncertain. The complication rates in emergent versus elective cholecystectomies are nearly twice as high, highlighting the need for improved surgical safety in this group [16].

This study aimed to compare intra- and postoperative complications and outcomes in patients undergoing laparoscopic cholecystectomy for acute cholecystitis using ultrasonic dissection or electrocautery dissection. It was conducted as a phase 3 trial following a phase 2b pilot study on the learning curve for ultrasonic fundus-first dissection in elective cholecystectomy [17, 18].

## Methods

### Study design

From 2019 to 2023, a randomized, parallel, multicentre, double-blinded, controlled trial was conducted across eight Swedish hospitals. The study was approved by the Regional Research Ethics Committee in Stockholm, Sweden (2016/1434–31/4, 2018/2587–32). The study report was structured under the CONSORT reporting guidelines [19].

### Participants

#### Patients

Eligible participants were patients ≥ 18 years old, diagnosed with acute cholecystitis according to the Tokyo guideline criteria [20] with a symptom duration of ≤ 7 days. Exclusion criteria were (1) American Society of Anaesthesiologists (ASA) physical status classification score of ≥ 4, (2) severe cholecystitis (Grade III as per the Tokyo guidelines) [20], (3) previous major upper abdominal surgery, (4) preoperative drainage of the gallbladder, (5) other acute or chronic abdominal diseases (e.g., pancreatitis, cirrhosis or hepatitis) with elevated liver enzymes, (6) pregnancy or (7) the inability to understand written instructions in Swedish. Patients were recruited before surgery. Oral and written informed consent was retrieved from all participants.

#### Surgeons

All participating surgeons were specialists or last year fellows in general surgery with previous experience with electrocautery and ultrasonic dissection. Experience from ultrasonic dissection was verified by inclusion in the pilot study [18], with surgeons performing ≥ 15 operations with the ultrasonic device or by video assessment.

### Procedures

The duration of symptoms in days, previous biliary colic, and the severity grade of cholecystitis [20], were registered upon inclusion. The participants were given a diary to evaluate the level of pain and nausea before and after the operation. They were also instructed to track their intake of pain medications and complete quality-of-life questionnaires (EQ-5D-5L) [21].

The operation was performed as early as the local circumstances allowed. The operating surgeon completed an electronic case report form (eCRF). All patients were registered in the Swedish Registry of Gallstone Surgery and Endoscopic Retrograde Cholangiopancreatography (GallRiks) [22]. It has a national coverage of 94.5% with a 97% follow-up frequency [16] and data have consistently shown high accuracy in reporting serious adverse events [23]. Patients were postoperatively treated according to local routines. Antibiotics were not routinely administered but were prescribed by the surgeon when indicated. Thrombosis prophylaxis was given to patients with risk factors for thrombotic events or extended operating times. Laboratory tests of red and white blood cell count, C-reactive protein and liver function tests were registered preoperatively, 24 h after surgery or earlier if the patient was discharged. Patients continued to fill out the diary for 7 days. Intra- and postoperative complications were retrieved from the eCRF and GallRiks, including a 30-day follow-up based on medical records. A telephone follow-up was registered by a research nurse at the principal study site 30 days after surgery. The eCRFs were periodically evaluated to identify any incorrect registrations, and the principal investigator was accessible to address inquiries throughout the study.

### Surgical intervention

Anaesthesia was conducted according to local routines. A standardized surgical technique specified in the study protocol was used with an open access technique (Hasson) below the umbilicus, followed by a standard four-port setting. Local anaesthetics were administered at all incision sites before the trocars were placed. Intra-abdominal pressure was kept at 12 mmHg or 15 mmHg in selected patients. For ultrasonic dissection, Harmonic HD1000i Shears™ (Ethicon Endosurgery [Europe] GmbH, Norderstedt, Germany) was used, set at level 3/5. The hospital’s monopolar electrocautery hook device was used for dissection, set to blend mode at 25W. The surgeon was allowed to choose the most suitable direction of dissection based on anatomical variations, the extent of inflammation and personal preference. Dissection was continued in both arms until a critical view of safety was achieved [24]. An intraoperative cholangiography was performed according to the routine in Sweden [25]. Intraoperative endoscopic removal of choledocholithiasis was recommended if common bile duct stones were encountered [26]. The cystic duct was divided with two clips on the proximal end. The division of the cystic artery was accomplished either by using clips or with the assistance of the ultrasonic device. A retrieval bag was used to extract the gallbladder.

### Outcomes

The primary endpoint was the total complication rate, comprising all intra- and postoperatively registered complications during the first 30 postoperative days. Secondary outcomes were operating time, conversion to open surgery, length of hospital stay, readmission and use of haemostatic agents.

### Randomization and blinding

After induction of anaesthesia, participants were randomly assigned to electrocautery dissection or ultrasonic dissection in a 1:1 allocation. The randomization was performed by the surgeon in a secure web-based randomization platform administered by The Information and Communication Technology Services and System Development at Umeå University, Sweden. A randomization sequence was created using a computer-generated algorithm with permuted blocks of variable sizes (4–6), stratified by centre. Until the inclusion process was finalized, the centre-specific allocation sequences were stored and accessible exclusively to the system developer. The allocation of study arms was concealed from the patient, as well as postoperative care providers and during the follow-up. No information about the allocated instrument was noted in the medical records but could be revealed for security reasons. The option of cross-over or conversion to open surgery was permitted, with documentation in the eCRF, if the surgeon deemed the allocated instrument unsafe due to inflammation or anatomical variations.

### Statistical analyses

The power calculation was based on results from the phase 2b trial [18], the annual GallRiks report from 2018 [27] and clinical results from a Swedish centre specialized in ultrasonic dissection [28]. A reduction in the total complication rate from 15% with electrocautery dissection to 5% was estimated. To detect a significant difference with a power of 80% at the *p* < 0.05 level, 141 patients would be needed in each group. We intended to include 300 patients to accommodate dropouts and patients lost to follow-up. No interim analysis was performed as both techniques are well-established and used routinely in Sweden. Differences between the two groups were analysed using the Pearson chi-square test for categorical variables and the independent t-test or the Mann–Whitney test for continuous variables. The intention-to-treat approach was used to analyse primary and secondary outcomes. Sex and ASA grade were included as confounders in the outcome analyses to address an uneven randomization. To avoid bias from clustering of procedures performed by individual surgeons, the primary outcome was analysed using logistic generalized estimated equations (GEEs). With risk difference (RD) for treatment outcome, with 95% confidence intervals (CIs) as measures of risk. Secondary outcome analysis was performed with a similar GEE model, an independent t-test, or the Mann–Whitney test as appropriate. No data monitoring committee was involved in overseeing the data. A two-sided *P*-value of < 0.05 was considered significant. Statistical analysis was performed with SPSS® version 28.0 (Armonk, NY, USA, IBM Corp.).

## Results

**Fig. 1:**
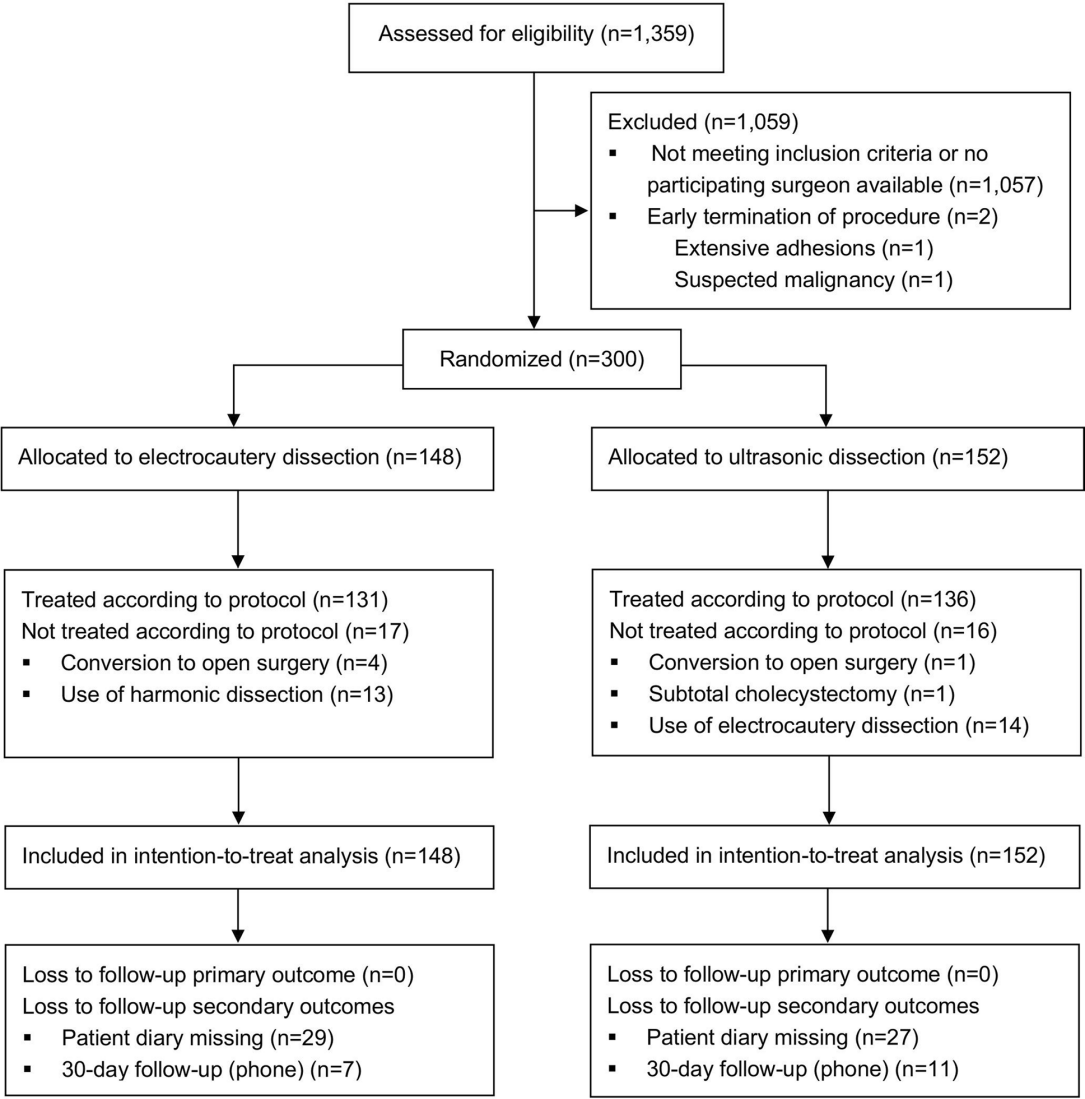
CONSORT flowchart of included patients

Between September 30, 2019, and March 22, 2023, 1359 patients who met the eligibility criteria were identified at the eight participating hospitals. Because only a few surgeons at each hospital participated in the study, patients were only recruited when these surgeons were available. Two patients were excluded intraoperatively due to incomplete cholecystectomy, with one presenting extensive adhesions and one having a suspected malignancy. Of the eligible patient population, 300 were randomly assigned to treatment. In total 148 patients assigned to electrocautery dissection and 152 to ultrasonic dissection were included (Fig. 1). The study cohort was older with equal sex distribution, compared to the excluded cohort (Supplementary Table 1). The operations were performed by 25 surgeons with a median of seven procedures per surgeon (Range 1–38). Patients assigned to ultrasonic dissection were more often of male sex with a higher ASA classification (Table 1). Only patients with an ASA grade of ≤ 3 were included, however, two patients allocated to ultrasonic dissection were intraoperatively graded as ASA 4 by the anaesthesiologist. In addition, patients who underwent ultrasonic dissection were more often preoperatively diagnosed with moderate cholecystitis (Grade II) and were intraoperatively found to have advanced cholecystitis (Table 2). At inclusion, 163 (54%) patients had no history of gallstone-related symptoms, and 21 (7.0%) had a documented history of cholecystitis. The mean duration of symptoms was 3 ± 1.5 days (Table 1). Dissection from Calot’s triangle and upwards were most common, but the fundus-first approach was more often used with ultrasonic dissection (17.1% vs. 2.0%) (Table 2).

**Table 1:** Demographics of the study patients

Patient characteristics	N (%)	N (%)
	Electrocautery dissection (n = 148)	Ultrasonic dissection (n = 152)
*Sex*		
Male	68 (45.9)	87 (57.2)
Female	80 (54.1)	65 (42.8)
*Age, years*		
< 25	3 (2.0)	1 (0.7)
25–49	35 (23.6)	34 (22.4)
50–74	89 (60.1)	87 (57.2)
≥ 75	21 (14.2)	30 (19.7)
*ASA grade*		
1	49 (33.1)	35 (23.0)
2	81 (54.7)	81 (53.3)
2–3	18 (12.2)	34 (22.4)
4	N/A	2 (1.3)
5	N/A	N/A
BMI (Mean, range) *	29 (18—43)	30 (18—52)
Missing	7 (4.7)	9 (5.9)
Previous cholecystitis	9 (6.1)	12 (7.9)
Duration of symptoms**		
1	23 (15.5)	16 (10.5)
2	26 (17.6)	35 (23.0)
3	25 (16.9)	46 (30.3)
4	23 (15.5)	21 (13.8)
5	19 (12.8)	11 (7.2)
> 5	12 (8.2)	6 (4.0
Missing	20 (13.5)	17 (11.2)
Preoperative grade of cholecystitis (Tokyo guidelines, TG) ^*^		
Mild, TG 1	60 (40.5)	56 (36.8)
Moderate, TG 2	85 (57.4)	96 (63.2)

*BMI, body mass index **Not a GallRiks variable, only available in included patients

**Table 2:** Surgery-related variables

	N (%)	N (%)
	Electrocautery dissection (n = 148)	Ultrasonic dissection (n = 152)
*Time of surgery*		
Daytime	79 (53.4)	70 (46.1)
Week time evening/night	42 (28.4)	48 (31.6)
Weekend	26 (17.6)	34 (22.4)
Missing	1 (0.7)	N/A
*Surgical access*		
Laparoscopic	144 (97.3)	151 (99.3)
Converted	4 (2.7)	1 (0.7)
*Direction of dissection*		
From Calot’s and up	128 (86.5)	89 (58.6)
Fundus first	3 (2.0)	26 (17.1)
Mixed technique	17 (11.5)	37 (24.3)
Level of cholecystitis		
*Accidental gallbladder perforation*		
Yes	77 (52.0)	87 (57.2)
No	71 (48.0)	65 (42.8)
*Voluntary bile aspiration*		
Yes	93 (62.8)	98 (64.5)
No	53 (35.8)	54 (35.5)
Missing	2 (1.4)	
*Antibiotics*		
Yes, prophylaxis	65 (43.9)	49 (32.2)
Yes, treatment	51 (34.5)	65 (42.8)
No	32 (21.6)	38 (25.0)
*Thrombosis prophylaxis*		
Yes	86 (58.1)	92 (60.5)
No	62 (41.9)	60 (39.5)
*Intraoperative findings (several options possible)*		
Mild cholecystitis	36 (24.3)	28 (18.4)
Advanced cholecystitis	81 (54.7)	93 (61.2)
Emphysematous cholecystitis	0	4 (2.6)
Gangrene	26 (17.6)	44 (28.9)
Liver abscess	1 (0.7)	1 (0.7)
Pericholecystic abscess	2 (1.4)	9 (5.9)
Spontaneous gallbladder perforation	8 (5.4)	8 (5.3)
Bile peritonitis	3 (2.0)	0
Acute cholecystitis with chronic signs	34 (23.0)	31 (20.4)
Acalculous cholecystitis	1 (0.7)	2 (1.3)
Missing	1 (0.7)	N/A

### Primary outcome

The total complication rate was 27 (18.2%) in patients assigned to electrocautery dissection, with 2 (1.4%) suffering an intraoperative and 26 (17.1%) a postoperative complication. The corresponding data in patients assigned to ultrasonic dissection was 26 (17.1%), with 2 (1.3%) intraoperative and 25 (16.4%) postoperative complications. The adjusted total risk of complications was 18.3% (95%CI, 13.0% to 25.1%) for electrocautery and 16.7% (95%CI, 11.7% to 23.2%) for ultrasonic dissection (RD of 1.6% (95%CI, − 7.2% to 10.4%, *P* = 0.720). The Clavien-Dindo (CD) classification [29] was used to assign the highest score in cases of multiple postoperative complications (Table 3). Postoperative complications with CD > 3 were more common for electrocautery (Table 3). The bile duct injury in the electrocautery group can be attributed to a complicated cholangiography and a perforating catheter. The ultrasonic device was used as a complement in 13 (8.8%) patients assigned to the electrocautery dissection arm, mainly due to highly vascularized gallbladders with extensive inflammation. In the ultrasonic dissection group electrocautery was used as a supplementary measure in 14 patients (9.2%), in most cases to achieve a more precise dissection within Calot’s triangle. One patient (0.7%) underwent subtotal cholecystectomy.

**Table 3:** Outcomes

	Electrocautery dissection (n = 148)	Ultrasonic dissection (n = 152)
	N (%)	N (%)
Total complications*	27 (18.2)	26 (17.1)
Intraoperative complications*	2 (1.4)	2 (1.3)
Bleeding—liver	1	1
Bile duct injury	1	
Bile leakage—liver		1
Postoperative complications*	26 (17.6)	25 (16.4)
Bile leakage—cystic	3	2
Bile obstruction—CBDS	2	1
Pancreatitis	4	3
Renal failure		2
Respiratory failure	1	1
Infection—Pneumonia	2	1
Infection—Deep abscess	2	4
Infection—Superficial wound	4	
Infection—Urinary tract		1
Infection—Unspecified	1	4
Deep venous thrombosis (DVT)		2
Thrombophlebitis		2
Urinary retention	4	2
Medical reaction	3	
*Postoperative complications stratified according to Clavien-Dindo*		
Grade I	3	1
Grade II	13	19
Grade IIIa (intervention without anaesthesia)	5	3
Grade IIIb (intervention with anaesthesia)	5	2
Grade IV		
Grade V		
*Operating time, Mean (range)*	100 min (26–215)	99 min (31–270)
*Bleeding, Mean (range)*	99 ml (0–500)	91 ml (0–500)
*Postoperative hospital stays, Mean (range)*	2.3 (0–23)	1.9 (0–10)
*Readmission 30 days, N (%)*	6 (4.1)	7 (4.6)

*Number of patients with ≥ 1 complications. Only the complication with the highest CD grade is presented

### Secondary outcomes

The mean operating time was 100 min (min) ± 38 for electrocautery and 99 min ± 42 for ultrasonic dissection (mean difference 1 min (95%CI − 8 min to 10 min, *P* = 0.816)). Four patients (2.7%) assigned to electrocautery dissection and one (0.7%) to ultrasonic dissection underwent conversion to open surgery (adjusted RD of 1.8% (95%CI − 0.8 to 4.4, *P* = 0.166)). The indications for conversion included advanced cholecystitis with atypical anatomy in three patients, advanced adhesions resulting from prior lower abdominal surgery in one patient and advanced cholecystitis with difficult anatomy due to obesity in one patient. The median postoperative stay was 2 days (IQR 1–2 days, range 0–23 days) for electrocautery and 1 day (IQR 1–2 days, range 0–10 days) for ultrasonic dissection (*P* = 0.191). No difference was seen in readmission rates between the groups (Table 3). The median estimated bleeding with electrocautery dissection was 60 ml (IQR 25–100 ml), and 50 ml (IQR 20–100 ml, *P* = 0.312) for ultrasonic dissection. Haemostatic agents were required in 40 (27.0%) patients assigned to electrocautery and 27 (17.8%) to ultrasonic dissection (adjusted RD 10.6% (95%CI, 1.3% to 19.8%, *P* = 0.025)).

### Other analyses

Accidental perforation of the gallbladder during dissection occurred in 77 patients (52.0%) who underwent electrocautery and in 87 patients (57.2%) who underwent ultrasonic dissection (*P* = 0.364). The gallbladder was intentionally punctured by the surgeon in 93 patients (62.8%) in the electrocautery group and 98 (64.5%) in the ultrasonic dissection group with gallbladder distention and difficulty in grasping listed as the most common causes. The cystic artery was ligated with clips in 115 patients (77.7%) who underwent electrocautery and 84 patients (54.9%) who underwent ultrasonic dissection. The ultrasonic dissector was used to ligate the artery in 54 patients (35.5%) in the latter group. No significant difference in the use of thrombosis prophylaxis was demonstrated (Table 2). A successful cholangiography was performed in 145 patients (98.0%) assigned to electrocautery and 141 (92.8%) allocated to ultrasonic dissection, with detection of common bile duct stones in 14 patients (9.5%) who underwent electrocautery dissection and 21 (13.8%) operated with ultrasonic dissection. Intraoperative ERCP was the most frequent method for stone removal, used in 9 patients (64.3%) allocated to electrocautery and 16 (76.2%) allocated to ultrasonic dissection. There was no discernible connection between the fundus-first approach and the increased rate of CBDS during ultrasonic dissection.

## Discussion

This multicentre RCT shows that ultrasonic dissection is a safe alternative to electrocautery dissection in laparoscopic cholecystectomies for emergency surgery patients with acute cholecystitis. The intra- and postoperative complication rates were comparable in both groups, suggesting that the techniques are safe for patients with mild-to-moderate acute cholecystitis. Patients randomized to ultrasonic dissection were predominantly male sex with higher ASA classification and exhibited a more advanced stage of inflammation. The presence of male sex and the extent of inflammation are known risk factors for complex procedures and the need for conversion to open surgery [30, 31]. Despite this, ultrasonic dissection reduced the use of additional haemostatic agents, although no significant difference in estimated blood loss was observed. Furthermore, a trend was noted among surgeons to prefer ultrasonic dissection over electrocautery in patients with extensive inflammation. This suggests that ultrasonic dissection may serve as a complement to electrocautery in cases of advanced inflammation prior to conversion to open surgery, or as a first-line approach in patients with complicated acute cholecystitis.

No significant reduction in operating time, hospital stays or gallbladder perforations was seen, which has been found for ultrasonic dissection in elective surgery [1–11]. The gallbladder perforation rates were high and slightly exceeded those reported in a Swedish observational study, which demonstrated a perforation rate of 48% in acute cholecystitis [32]. However, the higher rates align with expectations for a randomized controlled trial focusing on adverse outcomes. The reduced need for haemostatic agents supports previous studies on acute cholecystitis, indicating less bleeding with ultrasonic dissection [15, 33]. However, in contrast to these studies, our findings did not indicate a significant reduction in estimated blood loss or conversions to open surgery, and the conversion rate was low overall. Intraoperative blood loss is challenging to measure accurately in laparoscopic surgery, and the amount of bleeding during acute cholecystectomies can vary significantly. Therefore, the use of haemostatic agents may serve as a reliable indicator of intraoperative bleeding in this context. The decreased need for haemostatic agents strengthens previous studies demonstrating reduced indirect and direct costs with ultrasonic dissection in elective surgery [34–36]. The challenges associated with instrument handling during the learning curve are often cited as another drawback of ultrasonic dissection. In the pilot study, we showed that the fundus first technique with ultrasonic dissection has a low complication rate for residents and specialists in the first 15 operations [18]. The fundus-first approach was used in the pilot study because it is the preferred method at Sweden´s leading center for ultrasonic dissection in gallbladder surgery [1, 2]. This technique has been linked to low complication rates, including a minimal incidence of bile duct injuries (0.07%) [28]. Consequently, the ultrasonic instrument and the fundus-first technique were closely associated during the initial study. However, surgeons in the pilot study still often preferred to start the dissection from the triangle of Calot to identify crucial structures [18]. Given the complex anatomy and advanced inflammation in acute cholecystitis, surgeons in this study were allowed to choose the direction of the dissection based on their intraoperative assessment. Crossover occurred in less than one-tenth of patients in each arm. For ultrasonic dissection, electrocautery was used when dissecting the triangle of Calot, where the ultrasonic instrument may be considered blunt. Conversely, in the electrocautery group, the ultrasonic dissector was used more extensively to separate the gallbladder from the liver in cases of advanced inflammation. Given that an intention-to-treat analysis was employed, the potential for underestimating the results cannot be ruled out. In the pilot study the older version of the instrument was used (Harmonic ACE + (Ethicon Endosurgery [Europe] GmbH, Norderstedt, Germany), which many surgeons considered too blunt when dissecting the structures within the Calot’s triangle. We used the slimmer and slightly curved instrument in this study (Harmonic HD1000i Shears™ (Ethicon Endosurgery [Europe] GmbH, Norderstedt, Germany).

The study’s strength lies in its design as a randomized, double-blinded, parallel-group controlled trial conducted at eight hospitals in Sweden, involving 25 surgeons from university clinics, regional hospitals, and county hospitals. The study was preceded by a phase 2b pilot study on 240 elective cholecystectomies to evaluate the technique and safety of the procedure [18]. Intraoperative randomization, concealment of the allocated treatment, blinding of patients, postoperative caregivers and follow-up personnel and intention-to-treat analysis mitigate the risk of systematic errors. The study is, however, not without limitations. Because only surgeons with previous experience in both techniques could operate, 1059 eligible patients were not included in the study. The power calculation was based on a hypothetical significant reduction in complication rates based on best available data at the time [27, 28]. Concerning the results of this study, the potential safety effects of the intervention are likely smaller, suggesting that the study may have been underpowered to detect differences. Still, the present study shows comparable safety outcomes between ultrasonic dissection and standard treatment with electrocautery dissection. Despite randomization, the ultrasonic group included a higher proportion of male patients with higher ASA classification and patients with more advanced cholecystitis. Most participating surgeons had more experience in electrocautery dissection. We cannot rule out the possibility that ultrasonic dissection when performed by highly skilled surgeons who have surpassed the initial learning phase, could have resulted in varying rates of complications. All registered complications were included in the outcome analysis, but it is unlikely that all complications can be attributed to the allocated instrument. The intraoperative findings were based on subjective observation and no objective measure of difficulty, such as the Parkland grading system, was employed [37].

Despite evidence supporting the superiority of ultrasonic dissection over electrocautery in elective cholecystectomies [1–11, 15, 33], the technique is still not implemented in general practice. The reluctance to apply the technique may stem from its association with the fundus-first approach and a potentially increased rate of severe vascular and bile duct injuries [38]. In addition, the unfavourable reputation could be attributed to a notable rise in bile duct injuries following the implementation of the laparoscopic technique, particularly when used as a second-line approach in complicated cases [39]. Ultrasonic dissection is technically somewhat different from electrocautery dissection, necessitating training to achieve mastery of the technique. Based on our experience from the pilot work and the current study, we recommend that the technique is practiced in elective and less complicated cholecystectomies before it is used in complicated cases. We deem it wise to uphold a critical view of safety, regardless of the direction of the dissection [24, 40]. Another concern with ultrasonic devices relates to increased instrumental costs. To fully evaluate the benefits of ultrasonic dissection in acute cholecystectomies, further studies should include cost analyses and patient-reported outcomes. The results highlight the challenges of addressing differences in adverse events within a randomised controlled trial in emergency surgery, particularly when involving a reasonable number of patients. Based on our findings, both instruments can be considered safe for use in patients with acute cholecystitis. However, whether ultrasonic dissection should become the standard approach, or remain an alternative is a question for future studies. Future research should aim to assess the long-term outcomes and cost-effectiveness of ultrasonic dissection compared to traditional techniques, which will aid in guiding clinical decision-making in the management of acute cholecystitis.

## Conclusion

This randomized controlled trial demonstrates that ultrasonic dissection and electrocautery dissection have comparable complication risks in emergency surgery patients with mild-to-moderate acute cholecystitis. Ultrasonic dissection can serve as an alternative to, or adjunct with, electrocautery dissection in laparoscopic cholecystectomy for acute cholecystitis.

## Supplementary Information

Additional file1

Additional file2

Additional file3

## Data Availability

The datasets used and analysed during the current study are available from the corresponding author on reasonable request.

## Abbreviations

GallRiks: Swedish Registry of Gallstone Surgery and Endoscopic Retrograde Cholangiopancreatography
ASA: American Society of Anaesthesiologists physical status classification score
eCRF: Electronic case report form
ERCP: Endoscopic retrograde cholangiopancreatography
GEE: Generalized estimated equations
RD: Risk difference
CI: Confidence interval
IQR: Interquartile range
CD: Clavien–Dindo classification
