# Supplementary material for: Ultrasonic dissection versus electrocautery dissection in laparoscopic cholecystectomy for acute cholecystitis: a randomized controlled trial (SONOCHOL-trial)
Source: World J Emerg Surg. 2024 Nov 13;19:34. doi: 10.1186/s13017-024-00565-4 (PMC11562708; doi:10.1186/s13017-024-00565-4)
Supplement: Supplementary file 1 — Additional file1 [file 13017_2024_565_MOESM1_ESM.docx]

| SUPPLEMENTARY TABLE 1, COMPARISON OF ELIGIBLE AND INCLUDED PATIENTS | | | |
| --- | --- | --- | --- |
|  | **N (%)** | **N (%)** | **N (%)** |
| **Patient characteristics** | **Eligible patients**  **(n=1073)** | **Electrocautery dissection**  **(n=148)** | **Ultrasonic dissection**  **(n=152)** |
| **Sex** |  |  |  |
| Male | 545 (50.8) | 68 (45.9) | 87 (57.2) |
| Female | 528 (49.2) | 80 (54.1) | 65 (42.8) |
| **Age, years** |  |  |  |
| <25 | 25 (2.3) | 3 (2.0) | 1 (0.7) |
| 25–49 | 290 (27.0) | 35 (23.6) | 34 (22.4) |
| 50–74 | 529 (49.3) | 89 (60.1) | 87 (57.2) |
| ≥75 | 229 (21.3) | 21 (14.2) | 30 (19.7) |
| **ASA-grade** |  |  |  |
| 1 | 281 (26.2) | 49 (33.1) | 35 (23.0) |
| 2 | 512 (47.7) | 81 (54.7) | 81 (53.3) |
| 2–3 | 263 (24.5) | 18 (12.2) | 34 (22.4) |
| 4 | 15 (1.4) | N/A | 2 (1.3) |
| 5 | 2 (0.2) | N/A | N/A |
| **BMI (Mean, range)** | 29 (16 - 49) | 29 (18 - 43) | 30 (18 - 52) |
| Missing | 152 (14.2) | 7 (4.7) | 9 (5.9) |
| **Previous cholecystitis** | 99 (9.2) | 9 (6.1) | 12 (7.9) |
