# Supplementary material for: Ultrasonic dissection versus electrocautery dissection in laparoscopic cholecystectomy for acute cholecystitis: a randomized controlled trial (SONOCHOL-trial)
Source: World J Emerg Surg. 2024 Nov 13;19:34. doi: 10.1186/s13017-024-00565-4 (PMC11562708; doi:10.1186/s13017-024-00565-4)
Supplement: Supplementary file 2 — Additional file2 [file 13017_2024_565_MOESM2_ESM.pdf]

STUDY PROTOCOL

Ultrasonic Activated Scalpel Versus Electrocautery Based Dissection in Acute Cholecystitis, a Randomized Controlled Trial (SONOCHOL-trial).

Clinical registration: ClinicalTrials.gov: NTC03014817

Version: 3.0, translated from original document in Swedish.

CONTENTS

CONTENTS 1
STUDY ORGANIZATION 2
BACKGROUND 3
HYPOTHESIS 4
STUDY DESIGN 4
PRIMARY ENDPOINT 4
SECONDARY ENDPOINTS 4
INCLUSION CRITERIA 4
EXCLUSION CRITERIA 5
STATISTICAL ANALYSIS PLAN 6
POWER CALCULATION 6
STATISTICAL METHODS 6
DATA MONITORING 6
STUDY CONDUCT 7
1. TRAINING 7
2. DIAGNOSIS 7
3. INCLUSION 8
4. BASELINE OBSERVATIONS 8
5. RANDOMIZATION 8
6. OPERATION 8
Anesthesia 8
Antibiotic Prophylaxis 8
Surgical Procedures 8
Thromboprophylaxis 9
Record Keeping 9
GallRiks 9
8. ASSESSMENT OF DIFFICULTY 10
9. POSTOPERATIVE CARE 10
10. DISCHARGE 10
11. FOLLOW-UP 10
OTHER INFORMATION 11
CASE RECORD FORM 11
SCREENING LOG 11
BIOBANK 11
INTERIM ANALYSIS 11
MATERIAL HANDLING 11
ETHICAL APPLICATION 11
FLOWCHART – SONOCHOL 12
REFERENCES 13

STUDY ORGANIZATION

Sponsor: Karolinska Institutet
Address: Karolinska Institutet, 171 77 Stockholm, Sweden.
Phone: +46 8-524 800 00

Principal investigator:

Gabriel Sandblom, associate professor
Department of Clinical Science and Education, Södersjukhuset, Karolinska Institutet, Stockholm, Sweden.
Phone: +46 8-61 623 62. gabriel.sandblom@ki.se

Investigators:

My Blohm. PhD Student
Department of Surgery, Mora hospital, Mora, Sweden
Phone: +46 250-49 30 00. E-mail: my.blohm@regiondalarna.se
Department of Clinical Science and Education, Södersjukhuset, Karolinska Institutet, Stockholm, Sweden

Johanna Österberg, MD PhD, Consultant surgeon
Department of Surgery, Mora hospital, 792 85 Mora, Sweden
Phone: +46 250-49 30 00. E-mail: johanna.osterberg@regiondalarna.se
Department of Clinical Science and Education, Södersjukhuset, Karolinska Institutet, Stockholm, Sweden

Project administrator:

Anna Nordin. Research nurse
Department of Surgery, Mora hospital, 792 85 Mora, Sweden

Funding: Regional Research Council Mid Sweden (RFR-980175), Centre for Clinical Research, Falun, Sweden (CKFUU-96329, CKFUU-9692) and the Swedish ALF agreement (Stockholm County, 20190336). Instrument costs were covered by each participating hospital. Hospital within Stockholm County could be reimbursed from funds allocated through the Swedish ALF agreement.

BACKGROUND

Acute cholecystitis is a common reason for hospitalization in Sweden and worldwide. The diagnosis is based on clinical examination, blood tests, and radiology. The severity of cholecystitis can be graded using the Tokyo Guidelines criteria, first published in 2007 (1-3). Most patients with acute cholecystitis have mild to moderate symptoms, and the recommended treatment is surgery with cholecystectomy during the acute hospital stay. The inflammation in the gallbladder often complicates the surgery, and the risk of complications and conversion increases with postponed surgery (4). Several studies show the benefits of early surgery, if possible, within three days after the onset of symptoms (4, 5). Nearly all cholecystectomies performed in Sweden are registered in the Swedish National Quality Register for Gallstone Surgery and ERCP, GallRiks, which was founded in 2005 (6).

Most cholecystectomies today are performed with a laparoscopic technique and conversion to open surgery if needed (6). To dissect tissue and blood vessels, most surgeons use electrocautery, with an instrument shaped as a hook. Traditionally, the dissection begins at the neck of the gallbladder with a careful dissection of the cystic duct and cystic artery to achieve a "critical view of safety", before dividing the structures (7). The gallbladder is thereafter separated from the liver. Another technique that has gained popularity in recent years is dissection with instruments that use ultrasound-based tissue coagulation, ultrasonic dissection. These instruments are routinely used by many surgeons in other abdominal procedures.

Some surgical clinics in Sweden routinely use ultrasonic dissection in both acute and elective gallbladder surgery. They often separate the gallbladder from the liver in the opposite direction, i.e., from the top down, a technique known as "fundus first." The benefits of ultrasonic dissection in elective surgery are well-documented, but in theory, it could be even more advantageous in acute cholecystitis, where the hemostatic effect and tissue sealing are particularly important due to the pronounced inflammation (8-13).

Before this study, we conducted a pilot study focusing on the learning curve for the ultrasonic dissection technique using the fundus first approach in elective cholecystectomies (the LEFFE study, clinicaltrials.gov NCT03154164) (14). The results showed that the technique is safe in the hands of experienced gallbladder surgeons with few complications. Many of the participating surgeons, who had previously primarily operated with a diathermy hook, experienced some disadvantages in separating the gallbladder from the fundus downwards. For instance, it can lead to too central dissection and uncertainty in identifying important structures. In the study, each surgeon performed 15 elective operations, which is a criterion for participating in this study on acute cholecystitis. The combined experiences from the LEFFE study and the literature are that the ultrasonic dissection is a very effective instrument in gallbladder surgery, perhaps especially in more complicated conditions with bleeding and cholecystitis, regardless of the direction of dissection (9-11, 15). Based on this experience the choice of dissection direction in this study will be left to the operating surgeon.

Patients with acute cholecystitis often require several days of hospitalization and sometimes sick leave, so focusing on the patient's best interest and healthcare resources is important. As the instrument is more expensive than a diathermy hook, many refrain from using it routinely today. If the surgical technique with ultrasonic dissection in acute cholecystitis proves to reduce the frequency of complications, operation time, hospital stay, use of hemostatic agents, and sick leave duration, there is much to gain in terms of both patient safety and health economics.

HYPOTHESIS

For patients with mild to moderate acute cholecystitis, surgery using ultrasonic dissection is superior to surgery using the traditional electrocautery in terms of intra and postoperative complications.

STUDY DESIGN

The study is designed as a randomized, parallel, double-blinded, multicenter controlled trial.

PRIMARY ENDPOINT

Intra and postoperative complications, according to the GallRiks definition (e.g., conversion, bleeding, bile leakage, reoperation, infection, and mortality).

SECONDARY ENDPOINTS

Patient-Related

- Postoperative quality of life, pain, and nausea
- Pre- and postoperative blood tests

Health Economic Aspects

- Operation time
- Length of hospital stay, readmission rate, and sick leave
- Need for hemostatic agents

INCLUSION CRITERIA

- A maximum of seven days of symptoms consistent with acute cholecystitis, with laboratory tests and radiology (ultrasound, CT, or MRI) confirming the diagnosis
- ASA classification I-III
- Age ≥18 years

EXCLUSION CRITERIA

Before Inclusion

- Inability to understand the provided information, e.g., due to cognitive impairment or language difficulties where an interpreter is not available
- Pregnancy
- Severe cholecystitis with multi-organ failure at admission, Grade III according to the Tokyo guidelines
- Previous upper abdominal surgery
- Pre operative gallbladder drainage
- Signs of other concurrent acute or pronounced chronic abdominal diseases (pancreatitis, hepatitis, and severe liver cirrhosis, etc.)
- ASA classification ≥ 4

After Inclusion

- If the patient changes their preoperative decision to participate in the study
- Postponed surgery, planned for an elective operation
- Absence of a surgeon with experience from the techniques
- Development of severe cholecystitis during hospitalization, Grade III according to the Tokyo guidelines
- Development of other surgical or medical conditions that increase the risks of surgery and anesthesia, preventing surgery during the same hospital stay (e.g., acute myocardial infarction or heart failure)

STATISTICAL ANALYSIS PLAN

Power Calculation

According to the 2016 GallRiks annual report, the overall complication rate (Clavien-Dindo grade 2 or higher) for surgery for acute cholecystitis nationwide was approximately 13%. Since the 30-day follow-up does not always have 100% coverage, 13% is esteemed as underestimated; thus, we estimate a complication rate of 15% (16). Assuming that ultrasonic dissection reduces the complication risk to one-third, 5%, 141 patients are required in each group to achieve a probability of detecting the difference with a significance level of $p<0.05$. To adjust for attrition, we plan to include 150 patients in each arm, i.e., a total of 300 patients.

Statistical Methods

Descriptive statistics will be used to summarize baseline characteristics of patients in each treatment group. Continuous variables will be presented as mean ± standard deviation or median (interquartile range), while categorical variables will be presented as frequencies and percentages.

The primary analysis will be performed on an intention-to-treat basis, comparing the frequency of intra and postoperative complications between the two treatment groups using logistic regression. To account for clustering of procedures performed by individual surgeons a logistic generalized estimated equations (GEEs) might be used. Results will be presented as risk differences (RD) for treatment outcome, along with 95% confidence intervals (CIs).

Secondary outcome analysis will also be performed using a GEE model, or the independent t-test or Mann-Whitney test where appropriate.

A two-sided p-value < 0.05 will be considered statistically significant for all analyses.

DATA MONITORING

No data monitoring committee will be involved in overseeing the data.

STUDY CONDUCT

OVERVIEW OF STUDY CONDUCT (for details, see each point below)

1. **Training:** All participating surgeons must have undergone training and achieved the study-specified criteria.
2. **Diagnosis:** Patients with mild to moderate radiologically verified acute cholecystitis can be included in the study. A screening log will be kept for all patients scheduled for surgery.
3. **Inclusion:** Patients who meet the inclusion criteria will be asked to participate.
4. **Baseline Observations:** The patient receives a patient diary where the degree of pain and nausea is noted before the operation and during the first seven postoperative days. A quality-of-life questionnaire is completed preoperatively and after seven days. Laboratory tests are recorded before and after the operation.
5. **Randomization:** Occurs after anesthesia in the operating room to either diathermy dissection or ultrasonic dissection.
6. **Operation:** The operation is performed as soon as possible after hospital admission.
7. **GallRiks:** All patients are registered in GallRiks.
8. **Grading of inflammation:** The surgeon rates the level of inflammation.
9. **Postoperative Care:** The patient and postoperative staff remain blinded to the outcome of the randomization. Blood tests are conducted the day after the operation. The patient continues to fill out their diary.
10. **Discharge:** The patient is discharged home when deemed appropriate.
11. **Follow-up:** The patient receives a follow-up phone call with the research nurse at the responsible study site approximately four weeks after the operation. The diary is collected before this.

1. Training

Participating surgeons must be specialists or residents with experience in gallbladder surgery. The surgeon must have performed at least 15 operations with each technique. This can be verified through participation in the pilot study (LEFFE trial), or by the surgeon submitting a video for review and certifying that the requirement has been met. Those who do not meet these requirements cannot participate in the study for safety reasons.

2. Diagnosis

The diagnosis of acute cholecystitis is obtained through anamnesis, clinical findings, blood tests, and radiology (ultrasound, MRI, or CT) according to the Tokyo criteria (Appendix 1). The duration of symptoms is registered, and in case of uncertainty the shorter duration applies. The Tokyo criteria are used to grade the severity of the cholecystitis, with patients having mild to moderate cholecystitis being eligible for the study. A screening log is kept for all patients undergoing surgery.

3. Inclusion

The patient is informed by the admitting physician or the responsible surgeon, who provides information and obtains written informed consent. The information and a signed copy of the consent form must be shared with the patient.

4. Baseline Observations

The patient receives a diary for preoperative grading of perceived pain and nausea the first postoperative seven days. Quality of life is noted according to a validated protocol (EQ-5D-5L) (16, 17) preoperatively and after seven days. The diary is returned to Mora Lasarett in a provided envelope. Hb, WBC, CRP, and liver tests (ASAT, ALAT, ALP, Bilirubin, and Amylase) are recorded before the operation and 20-24 hours after, or before discharge if the patient is discharged earlier.

5. Randomization

Randomization to diathermy dissection or ultrasonic dissection takes place in the operating room when the patient is anesthetized using a study-specific electronic randomization module.

6. Operation

Anesthesia

Anesthesia, preoperative pain relief, and antiemetics are administered according to local routines.

Antibiotic Prophylaxis

Not routinely given but prescribed if needed.

Surgical Procedures

Access to the abdomen is achieved using an open technique at the umbilicus as the standard method. If this is difficult, a Veress needle can be used under the left costal arch, with an optical trocar as the first port. The surgery is performed with an intra-abdominal pressure of 12 mm Hg; this pressure can be increased to 16 mm Hg if necessary, which is noted in the protocol (e.g., BMI > 35). Standard port placement with four ports is used: a 12-mm port just below the umbilicus, a 12-mm port in the epigastrium under the xiphoid process, and two 5-mm ports under the right costal arch. Local Anesthesia is administered with 7.5% Ropivacaine before making the incisions. A 30-degree 12 mm standard optic is used. If the gallbladder is severely distended, it may be drained of bile to facilitate dissection.

When using diathermy dissection, a monopolar diathermy hook with a blend of cutting and coagulation at 25 W is used. Ultrasonic dissection is performed with an ultrasonic shear (Harmonic HD 1000i Shears, Ethicon Endosurgery, Norderstedt, Germany). Both techniques aim to achieve a satisfactory “critical view of safety.” Before dividing the cystic duct, an intraoperative cholangiography is performed to ensure and map the anatomy and identify any stones. For stones in the common bile duct, an intraoperative ERCP with rendezvous technique or trans cystic stone extraction is performed. If competence for intraoperative stone removal is lacking, adequate preparations for postoperative ERCP are made by placing a guidewire or stent.

After cholangiography, the cystic duct is divided with clips: one near the gallbladder and two remaining on the cystic duct stump. The cystic artery is divided with a clip, diathermy, or ultrasonic shear, and this must be noted in the protocol. The gallbladder is separated from the liver bed and removed using a retrieval bag via the umbilical port. The main direction of dissection is adapted to the anatomy and the surgeon's preference. Bile and blood from the surgical area are aspirated, and the abdominal cavity is flushed clean, leaving a maximum of 100 ml of fluid.

The fascia below the umbilicus is closed with a continuous slowly absorbable suture, and the skin is closed with absorbable sutures. Dressing is applied according to local routines. Pathological examination is sent if needed. Antibiotics are not routinely given but can be prescribed by the responsible surgeon if indicated.

Thromboprophylaxis

Is ordered by the surgeon, based on the patient's risk factors according to local routines.

Record Keeping

In the operation report, the choice of instruments should not be specified; however, it should be noted that the patient is operated on within the framework of the SONOCHOL study. The code can be broken locally at the clinic or by the research nurse in Mora if necessary.

GallRiks

The operation is registered as per routine in GallRiks immediately following the operation, where a complete 30-day follow-up is mandatory. The 30-day follow-up is crucial for the study as it provides patient characteristics, complication rates, antibiotic treatment, thromboprophylaxis, mortality, etc. The application of interest and project application for data extraction has been submitted and approved by the GallRiks board.

8. Assessment of Difficulty

The difficulty in performing the cholecystectomy is evaluated using a VAS scale graded from 1-100, where three procedural steps are scored, and an overall assessment. The aspects evaluated and scored are:

1. Identification of the gallbladder and dissection from surrounding tissue.
2. Dissection and exposure of Calot's triangle and separation of the gallbladder from the liver bed.
3. Conducting an intraoperative cholangiography.
4. The operation as a whole.

9. Postoperative Care

Patients, as well as staff involved in postoperative care, remain blinded to the outcome of the randomization. If the patient wishes, the randomization outcome can be disclosed after the telephone follow-up at least four weeks post-operation. In the first seven days following the operation, patients indicate the level of pain and nausea in the diary using a VAS scale ranging from 1 (no pain or nausea) to 10 (maximum discomfort). After seven days, a quality of life questionnaire (EQ-5D-5L) is also completed. Blood tests including Hb, WBC, CRP, and liver function tests are conducted 20-24 hours postoperatively or before discharge if the patient is discharged earlier. Postoperative pain relief is primarily provided with Paracetamol 500 mg, Diclofenac 50 mg, and if stronger medication is needed, Oxynorm 5 mg. The number of tablets taken per day is recorded by the patient in the patient diary, with the option for additional notes and comments.

10. Discharge

The patient is discharged home when deemed appropriate by the responsible physician. In most cases, a one-week recovery period is sufficient, so sick leave is generally not recommended.

11. Follow-Up

One month after discharge, the patient has a scheduled telephone follow-up with the study coordinator in Mora. During this call, any complications and duration of sick leave are noted. The patient diary should have been submitted before this call.

OTHER INFORMATION

Case Record Form

An inclusion sheet is filled out at the time of inclusion and at completion. Operation-related parameters are entered by the operator into a specific electronic form designed for the study, closely following the operation. Other parameters are obtained from attached lab results, GallRiks, and the patient diary.

Screening Log

A screening log should be maintained at the clinic throughout the study. It records all patients undergoing emergency surgery for acute cholecystitis, whether they were included, and the reasons for any exclusion. The number of eligible patients will be retrieved from GallRiks at the end of the study.

Biobank

All laboratory tests in the study are included in the standard routine during the treatment and management of patients with acute cholecystitis. Blood samples will therefore not be stored in a biobank.

Interim Analysis

As both techniques are well-established and used daily in Sweden, no interim analysis is planned.

Material Handling

Personal identification numbers and study IDs are noted on an inclusion sheet and in a screening log. These are safely stored and the inclusion sheets are sent fully completed, along with copies of consent forms, to the study coordinator in Mora. Consent forms are stored long-term at the operating hospital or alternatively at Mora Hospital. Copies of all documents should be retained at the clinic as a safeguard until the study is completed. Other materials are stored long-term in KI-ELN (Karolinska Institutet's electronic lab notebook).

Ethical Application

An ethical application has been submitted and approved by the Stockholm Ethics Review Board with the reference number 2016/1434-31/4, with a supplementary application 2018/2587-32.

FLOWCHART – SONOCHOL

Inclusion Criteria
Patients with radiographically confirmed acute cholecystitis with symptoms ≤7 days.

Exclusion Criteria

- Inability to understand the provided information.
- History of symptoms for more than 7 days.
- Age < 18 years.
- Severe cholecystitis with multiple organ failure.
- Signs of other concurrent acute or chronic abdominal diseases.
- ASA (American Society of Anesthesiologists) classification ≥ 4.

↓

Written informed consent is obtained before surgery.

An inclusion form is filled out, and a **Patient Diary** is given to the patient.

↓

The patient undergoes surgery as soon as possible after admission. **Randomization** is done under anesthesia to:

- Electrocautery dissection
- Ultrasonic dissection

Blood sampling for hemoglobin (Hb), leukocytes (WBC), C-reactive protein (CRP), and liver tests is performed pre- and post-operatively.

→

The randomization module can be found at the link: www.norrlandskirurgi.se

Password: Provided to each clinic at study start

The randomization number is noted in the patient's medical record, not the chosen instrument.

↓

Electronic Case Report Form (CRF) and **GallRiks** are filled out by the operator.
Study ID (inclusion form) and **randomization number** are recorded.

→

Follow-up

The Patient diary is sent in after 7 days in a pre-stamped envelope.

Telephone follow-up is conducted by a research nurse after approximately 4 weeks.

A screening log is kept at the clinic. This is sent with the inclusion form, lab results, and consent form by registered mail to:

My Blohm, SONOCHOL-studien, Kirurgkliniken, Mora lasarett, 792 85 Mora

REFERENCES

1. Hirota M, Takada T, Kawarada Y, Nimura Y, Miura F, Hirata K, et al. Diagnostic criteria and severity assessment of acute cholecystitis: Tokyo Guidelines. J Hepatobiliary Pancreat Surg. 2007;14(1):78-82.
2. Yokoe M, Takada T, Strasberg SM, Solomkin JS, Mayumi T, Gomi H, et al. New diagnostic criteria and severity assessment of acute cholecystitis in revised Tokyo Guidelines. J Hepatobiliary Pancreat Sci. 2012;19(5):578-85.
3. Yokoe M, Hata J, Takada T, Strasberg SM, Asbun HJ, Wakabayashi G, et al. Tokyo Guidelines 2018: diagnostic criteria and severity grading of acute cholecystitis (with videos). J Hepatobiliary Pancreat Sci. 2018;25(1):41-54.
4. Blohm M, Osterberg J, Sandblom G, Lundell L, Hedberg M, Enochsson L. The Sooner, the Better? The Importance of Optimal Timing of Cholecystectomy in Acute Cholecystitis: Data from the National Swedish Registry for Gallstone Surgery, GallRiks. J Gastrointest Surg. 2017;21(1):33-40.
5. SBU. Operation vid besvär av sten i gallblåsan och akut gallblåseinflammation. Stockholm: Statens beredning för medicinsk och social utvärdering (SBU). SBU-rapport nr 259. 2016.
6. Gallriks [Available from: http://www.ucr.uu.se/gallriks/.
7. Strasberg SM. Avoidance of biliary injury during laparoscopic cholecystectomy. J Hepatobiliary Pancreat Surg. 2002;9(5):543-7.
8. Cengiz Y, Janes A, Grehn A, Israelsson LA. Randomized trial of traditional dissection with electrocautery versus ultrasonic fundus-first dissection in patients undergoing laparoscopic cholecystectomy. Br J Surg. 2005;92(7):810-3.
9. Cengiz Y, Dalenback J, Edlund G, Israelsson LA, Janes A, Moller M, et al. Improved outcome after laparoscopic cholecystectomy with ultrasonic dissection: a randomized multicenter trial. Surg Endosc. 2010;24(3):624-30.
10. Catena F, Ansaloni L, Di Saverio S, Gazzotti F, Coccolini F, Pinna AD. Prospective analysis of 101 consecutive cases of laparoscopic cholecystectomy for acute cholecystitis operated with harmonic scalpel. Surg Laparosc Endosc Percutan Tech. 2009;19(4):312-6.
11. Catena F, Di Saverio S, Ansaloni L, Coccolini F, Sartelli M, Vallicelli C, et al. The HAC trial (harmonic for acute cholecystitis): a randomized, double-blind, controlled trial comparing the use of harmonic scalpel to monopolar diathermy for laparoscopic cholecystectomy in cases of acute cholecystitis. World J Emerg Surg. 2014;9(1):53.

12. Power C, Maguire D, McAnena OJ, Calleary J. Use of the ultrasonic dissecting scalpel in laparoscopic cholecystectomy. Surg Endosc. 2000;14(11):1070-3.
13. Sietses C, Eijsbouts QAJ, von Blomberg BM, Cuesta MA. Ultrasonic energy vs monopolar electrosurgery in laparoscopic cholecystectomy: influence on the postoperative systemic immune response. Surg Endosc. 2001;15(1):69-71.
14. Blohm M, Sandblom G, Enochsson L, Cengiz Y, Austrums E, Abdon E, et al. Learning by doing: an observational study of the learning curve for ultrasonic fundus-first dissection in elective cholecystectomy. Surg Endosc. 2022;36(6):4602-13.
15. Janssen IM, Swank DJ, Boonstra O, Knipscheer BC, Klinkenbijl JH, van Goor H. Randomized clinical trial of ultrasonic versus electrocautery dissection of the gallbladder in laparoscopic cholecystectomy. Br J Surg. 2003;90(7):799-803.
16. Enochsson L, Blohm M, Sandblom G, Jonas E, Hallerback B, Lundell L, et al. Inversed relationship between completeness of follow-up and coverage of postoperative complications in gallstone surgery and ERCP: a potential source of bias in patient registers. BMJ Open. 2018;8(1):e019551.
17. Herdman M, Gudex C, Lloyd A, Janssen M, Kind P, Parkin D, et al. Development and preliminary testing of the new five-level version of EQ-5D (EQ-5D-5L). Qual Life Res. 2011;20(10):1727-36.
18. EuroQol G. EuroQol--a new facility for the measurement of health-related quality of life. Health Policy. 1990;16(3):199-208.
